# Training of Peer Reviewers: Validation of a 5-Point Rating Scale

**DOI:** 10.1371/journal.pmed.0040166

**Published:** 2007-04-24

**Authors:** Michael Callaham

We regret that in our paper in the January issue of *PLoS Medicine* [1], we failed to cite an important recent study [2] that validates a simple 5-point quality rating score virtually identical to the one we used, and which we find more efficient than scores with multiple subscales. We apologize for the omission of this helpful research.
